# Supplementary material for: Lumbar Paravertebral Muscle Pain Management Using Kinesitherapy and Electrotherapeutic Modalities
Source: Healthcare (Basel). 2024 Apr 18;12(8):853. doi: 10.3390/healthcare12080853 (PMC11050304; doi:10.3390/healthcare12080853)
Supplement: Supplementary file 1 [file healthcare-12-00853-s001.zip › Supplementary File Table S1.pdf]

Table S1. Evolution of pain parameters and functional assessment.

	**VAS -AVG(SD)**			**LBP-M AVG(SD)**		
	T1-T2	T2-T3	T1-T3	T1-T2	T2-T3	T1-T3
G1 Group	8.19±0.45	5.59±0.71	3.58±0.75	8.74±0.89	16.25±1.41	25.35±1.34
G2 Group	7.72±0.62	5.59±0.69	3.61±0.78	8.81±1.29	16.23±1.77	24.15±2.02
