# Supplementary material for: Lumbar Paravertebral Muscle Pain Management Using Kinesitherapy and Electrotherapeutic Modalities
Source: Healthcare (Basel). 2024 Apr 18;12(8):853. doi: 10.3390/healthcare12080853 (PMC11050304; doi:10.3390/healthcare12080853)
Supplement: Supplementary file 1 [file healthcare-12-00853-s001.zip › Supplementary File Table S10.pdf]

Table S10. Evolution of physiological parameters HR and SAT O_2 in study batches.

	**HR- AVG(SD)**			**SAT O_2 - AVG(SD)**		
	**T1-T2**	**T2-T3**	**T1-T3**	**T1-T2**	**T2-T3**	**T1-T3**
G1 Group	88.78±14.25	75.29±15.79	77.05±10.39	96.37±0.94	97.51±0.75	97.63±0.73
G2 Group	76.09±11.23	77.63±13.37	70.31±7.76	96.37±0.58	96.74±0.65	96.56±0.67
