# Supplementary material for: Lumbar Paravertebral Muscle Pain Management Using Kinesitherapy and Electrotherapeutic Modalities
Source: Healthcare (Basel). 2024 Apr 18;12(8):853. doi: 10.3390/healthcare12080853 (PMC11050304; doi:10.3390/healthcare12080853)
Supplement: Supplementary file 1 [file healthcare-12-00853-s001.zip › Supplementary File Table S11.pdf]

Table S11. Evolution of physiological parameters in study batches.

	**SBP – AVG(SD)**			**DBP- AVG(SD)**		
	**T1-T2**	**T2-T3**	**T1-T3**	**T1-T2**	**T2-T3**	**T1-T3**
G1 Group	130.22±5.64	115.44±7.93	115.81±7.81	80.51±6.69	68.58±7.69	73.09±5.59
G2 Group	132.91±6.41	118.21±7.89	121.77±7.06	81.05±5.71	72.51±6.25	76.21±5.43
