# Supplementary material for: Lumbar Paravertebral Muscle Pain Management Using Kinesitherapy and Electrotherapeutic Modalities
Source: Healthcare (Basel). 2024 Apr 18;12(8):853. doi: 10.3390/healthcare12080853 (PMC11050304; doi:10.3390/healthcare12080853)
Supplement: Supplementary file 1 [file healthcare-12-00853-s001.zip › Supplementary File Table S2.pdf]

Table S2. Evolution of mobility and disability parameters.

	Oswestry Index - AVG(SD)			FFDI-AVG(SD)		
	**T1-T2**	**T2-T3**	**T1-T3**	**T1-T2**	**T2-T3**	**T1-T3**
G1 Group	34.89±9.54	24.73±9.78	17.25±8.25	24.89±2.03	15.54±0.63	8.05±0.82
G2 Group	38.64±7.29	31.28±7.51	24.5±7.25	23.72±0.99	21.21±1.21	19.53±1.99
