# Supplementary material for: Lumbar Paravertebral Muscle Pain Management Using Kinesitherapy and Electrotherapeutic Modalities
Source: Healthcare (Basel). 2024 Apr 18;12(8):853. doi: 10.3390/healthcare12080853 (PMC11050304; doi:10.3390/healthcare12080853)
Supplement: Supplementary file 1 [file healthcare-12-00853-s001.zip › Supplementary File Table S3.pdf]

Table S3. Evolution of physiological parameters in study batches.

	SBP- AVG(SD)			DBP- AVG(SD)			HR- AVG(SD)			SAT O_2- AVG(SD)		
	**T1-T2**	**T2-T3**	**T1-T3**	**T1-T2**	**T2-T3**	**T1-T3**	**T1-T2**	**T2-T3**	**T1-T3**	**T1-T2**	**T2-T3**	**T1-T3**
G1	130±5.5	116±7.6	116±7.5	80±6.9	70±7.9	73±6.5	83±10.7	75±11.6	76±7.9	97±1.1	98±6.8	98±0.8
G2	134±5.8	121±7.2	123±6.8	82±5.8	73±8.1	77±5.8	78±12.1	76±11.3	71±7.9	97±0.8	97±0.8	97±0.9
