# Supplementary material for: Lumbar Paravertebral Muscle Pain Management Using Kinesitherapy and Electrotherapeutic Modalities
Source: Healthcare (Basel). 2024 Apr 18;12(8):853. doi: 10.3390/healthcare12080853 (PMC11050304; doi:10.3390/healthcare12080853)
Supplement: Supplementary file 1 [file healthcare-12-00853-s001.zip › Supplementary File Table S4.pdf]

Tabel S4. Evolution of pain parameters and functional assessment.

	VAS- AVG(SD)			LBP-M- AVG(SD)		
	**T1-T2**	**T2-T3**	**T1-T3**	**T1-T2**	**T2-T3**	**T1-T3**
G1 Group	8.19±0.39	5.51±0.59	3.47±0.63	8.68±0.86	16.34±1.34	25.26±1.49
G2 Group	7.71±0.62	5.64±0.72	3.68±0.83	8.75±1.14	16.18±1.74	23.19±2.08
