# Supplementary material for: Lumbar Paravertebral Muscle Pain Management Using Kinesitherapy and Electrotherapeutic Modalities
Source: Healthcare (Basel). 2024 Apr 18;12(8):853. doi: 10.3390/healthcare12080853 (PMC11050304; doi:10.3390/healthcare12080853)
Supplement: Supplementary file 1 [file healthcare-12-00853-s001.zip › Supplementary File Table S5.pdf]

Tabel S5. Evolution of mobility and disability parameters.

	**Oswestry Index - AVG(SD)**			**FFDI- AVG(SD)**		
	**T1-T2**	**T2-T3**	**T1-T3**	**T1-T2**	**T2-T3**	**T1-T3**
G1 Group	36.17±9.73	26.09±9.98	18.49±8.43	25.21±1.98	15.49±0.74	8.12±0.54
G2 Group	38.21±7.21	31.05±7.35	24.41±7.21	23.84±0.94	21.27±1.21	19.43±2.18
