# Supplementary material for: Lumbar Paravertebral Muscle Pain Management Using Kinesitherapy and Electrotherapeutic Modalities
Source: Healthcare (Basel). 2024 Apr 18;12(8):853. doi: 10.3390/healthcare12080853 (PMC11050304; doi:10.3390/healthcare12080853)
Supplement: Supplementary file 1 [file healthcare-12-00853-s001.zip › Supplementary File Table S6.pdf]

Table S6. Evolution of physiological parameters HR and SAT O_2 in study batches.

	**HR- AVG(SD)**			**SAT O_{2}- AVG(SD)**		
	**T1-T2**	**T2-T3**	**T1-T3**	**T1-T2**	**T2-T3**	**T1-T3**
G1 Group	83.35±10.67	75.35±11.63	76.44±7.91	96.56±1.01	97.53±6.82	97.79±0.77
G2 Group	78.21±12.13	76.23±11.32	70.66±7.97	96.64±0.87	97.02±0.82	96.86±0.88
