# Supplementary material for: Lumbar Paravertebral Muscle Pain Management Using Kinesitherapy and Electrotherapeutic Modalities
Source: Healthcare (Basel). 2024 Apr 18;12(8):853. doi: 10.3390/healthcare12080853 (PMC11050304; doi:10.3390/healthcare12080853)
Supplement: Supplementary file 1 [file healthcare-12-00853-s001.zip › Supplementary File Table S7.pdf]

Table S7. Evolution of physiological parameters SBP and DBP in study batches.

	SBP- AVG(SD)			DBP- AVG(SD)		
	**T1-T2**	**T2-T3**	**T1-T3**	**T1-T2**	**T2-T3**	**T1-T3**
G1 Group	129.79±5.52	115.65±7.64	116.37±7.53	79.81±6.89	69.91±7.91	73.15±6.45
G2 Group	134.09±5.66	120.5±7.18	123.23±6.84	82.05±5.81	73.43±8.06	76.84±5.79
