# Supplementary material for: Lumbar Paravertebral Muscle Pain Management Using Kinesitherapy and Electrotherapeutic Modalities
Source: Healthcare (Basel). 2024 Apr 18;12(8):853. doi: 10.3390/healthcare12080853 (PMC11050304; doi:10.3390/healthcare12080853)
Supplement: Supplementary file 1 [file healthcare-12-00853-s001.zip › Supplementary File Table S8.pdf]

Table S8. Evolution of pain parameters and functional assessment.

	VAS- AVG(SD)			LBP-M- AVGSD)		
	**T1-T2**	**T2-T3**	**T1-T3**	**T1-T2**	**T2-T3**	**T1-T3**
G1 Group	8.19±0.51	5.68±0.82	3.71±0.84	8.81±0.93	16.15±1.49	25.46±1.16
G2 Group	7.74±0.62	5.58±0.66	3.61±0.69	8.88±1.45	16.26±1.82	24.42±1.97
