# Supplementary material for: Lumbar Paravertebral Muscle Pain Management Using Kinesitherapy and Electrotherapeutic Modalities
Source: Healthcare (Basel). 2024 Apr 18;12(8):853. doi: 10.3390/healthcare12080853 (PMC11050304; doi:10.3390/healthcare12080853)
Supplement: Supplementary file 1 [file healthcare-12-00853-s001.zip › Supplementary File Table S9.pdf]

Table S9. Evolution of mobility and disability parameters.

	Oswestry Index - AVG(SD)			FFDI- AVG(SD)		
	**T1-T2**	**T2-T3**	**T1-T3**	**T1-T2**	**T2-T3**	**T1-T3**
G1 Group	33.63±9.38	23.46±9.56	16.17±7.98	24.61±2.06	15.61±0.49	8.22±0.85
G2 Group	38.63±7.29	31.07±7.57	24.12±7.15	23.61±1.05	21.12±1.22	19.61±1.81
